# Correction: Individual Differences in Ethanol Locomotor Sensitization Are Associated with Dopamine D1 Receptor Intra-Cellular Signaling of DARPP-32 in the Nucleus Accumbens

**DOI:** 10.1371/journal.pone.0103667

**Published:** 2014-07-21

**Authors:** The PLOS ONE Staff

## Notice of Republication

This article was republished on June 27, 2014, to correct an error in the citation. The publisher apologizes for the error. Please download this article again to view the correct version. The originally published, uncorrected article and the republished, corrected article are provided here for reference.

## Supporting Information

File S1 Originally published, uncorrected article.
(PDF)

File S2 Republished, corrected article.
(PDF)
