# Correction: Individual Differences in Ethanol Locomotor Sensitization Are Associated with Dopamine D1 Receptor Intra-Cellular Signaling of DARPP-32 in the Nucleus Accumbens

**DOI:** 10.1371/journal.pone.0104151

**Published:** 2014-07-25

**Authors:** The PLOS ONE Staff

There is an error in the first sentence of the Introduction. The publisher apologizes for the error. The correct sentence is: Although alcoholism is a worldwide problem resulting in millions of deaths, only a small percentage of alcohol users become addicted [1].
