# Supplementary material for: Individual Differences in Ethanol Locomotor Sensitization Are Associated with Dopamine D1 Receptor Intra-Cellular Signaling of DARPP-32 in the Nucleus Accumbens
Source: PLoS One. 2014 Jun 11;9(6):e98296. doi: 10.1371/journal.pone.0098296 (PMC4053371; doi:10.1371/journal.pone.0098296)
Supplement: Figure S1 — Novelty response does not predict the development of ethanol locomotor sensitization. Locomotor activity (means ± S.E.M.) for 15 min in the novelty test when saline, non-sensitized (nsens) or sensitized (sens) groups were exposure to the locomotor activity cage for the first time without drug administrations of mice. (A) Cohort of mice (saline, n = 8; nsens, n = 5; sens, n = 8) that received the lower dose of SKF during the challenge phase. (B) Cohort of mice (saline, n = 11; nsens, n = 6; sens, n = 7) that received the higher dose of SKF during the challenge phase. (C) Cohort of mice (saline, n = 8; nsens, n = 8; sens, n = 9) that was designed for DARPP-32 measures after saline administration. (D) Cohort of mice (saline, n = 6; nsens, n = 7; sens, n = 7) that was designed for DARPP-32 measures after SKF administration. (DOCX) [file pone.0098296.s001.docx]

**Supplementary Material**

**Individual differences in ethanol locomotor sensitization are associated with dopamine D1 receptor intra-cellular signaling of DARPP-32 in the nucleus accumbens.**

Karina Possa Abrahao, PhD; Francine Oliveira Goeldner, PhD; Maria Lucia Oliveira Souza-Formigoni, PhD.

No differences were observed among saline, non-sensitized and sensitized mice in the novelty test (One Way ANOVA: FigS1A F(2,18) = 0.01; FigS1B F(2,21) = 0.60; FigS1C F(2,22) = 0.52; FigS1D F(2,19) = 0.56). Thus, the locomotor response to a new environment does not predict the variability of the development of behavioral sensitization.
