# Supplementary material for: Individual Differences in Ethanol Locomotor Sensitization Are Associated with Dopamine D1 Receptor Intra-Cellular Signaling of DARPP-32 in the Nucleus Accumbens
Source: PLoS One. 2014 Jun 11;9(6):e98296. doi: 10.1371/journal.pone.0098296 (PMC4053371; doi:10.1371/journal.pone.0098296)
Supplement: Material S1 — Statistical analyses of novelty response for each behavioral sensitization experiment. (DOCX) [file pone.0098296.s002.docx]

**Supplementary Material S1**

No differences were observed among saline, non-sensitized and sensitized mice in the novelty test (One Way ANOVA: FigS1A F(2,18) = 0.01; FigS1B F(2,21) = 0.60; FigS1C F(2,22) = 0.52; FigS1D F(2,19) = 0.56). Thus, the locomotor response to a new environment does not predict the variability of the development of behavioral sensitization.
