# Supplementary material for: Correction: Individual Differences in Ethanol Locomotor Sensitization Are Associated with Dopamine D1 Receptor Intra-Cellular Signaling of DARPP-32 in the Nucleus Accumbens
Source: PLoS One. 2014 Jul 21;9(7):e103667. doi: 10.1371/journal.pone.0103667 (PMC4105473; doi:10.1371/journal.pone.0103667)
Supplement: File S1 — Originally published, uncorrected article. (PDF) [file pone.0103667.s001.pdf]

Individual Differences in Ethanol Locomotor Sensitization Are Associated with Dopamine D1 Receptor Intra-Cellular Signaling of DARPP-32 in the Nucleus Accumbens

Karina Possa Abrahao¤, Francine Oliveira Goeldner, Maria Lucia Oliveira Souza-Formigoni*

Departamento de Psicobiologia, Escola Paulista de Medicina, Universidade Federal de São Paulo, SP, Brazil

Abstract

In mice there are clear individual differences in the development of behavioral sensitization to ethanol, a progressive potentiation of its psychomotor stimulant effect. Variability in the behavioral responses to ethanol has been associated with alcohol preference. Here we investigated if the functional hyperresponsiveness of D1 receptors observed in ethanol sensitized mice leads to an increased activation of DARPP-32, a central regulatory protein in medium spiny neurons, in the nucleus accumbens - a brain region known to play a role in drug reinforcement. Swiss Webster mice received ethanol (2.2 g/kg/day) or saline i.p. administrations for 21 days and were weekly evaluated regarding their locomotor activity. From those treated with ethanol, the 33% with the highest levels of locomotor activity were classified as ''sensitized'' and the 33% with the lowest levels as "non-sensitized''. The latter presented similar locomotor levels to those of saline-treated mice. Different subgroups of mice received intra-accumbens administrations of saline and, 48 h later, SKF-38393, D1 receptor agonist 0.1 or 1 µg/side. Indeed, sensitized mice presented functional hyperresponsiveness of D1 receptors in the accumbens. Two weeks following the ethanol treatment, other subgroups received systemic saline or SKF 10 mg/kg, 20 min before the euthanasia. The nucleus accumbens were dissected for the Western Blot analyses of total DARPP-32 and phospho-Thr34-DARPP-32 expression. D1 receptor activation induced higher phospho-Thr34-DARPP-32 expression in sensitized mice than in non-sensitized or saline. The functionally hyperresponsiveness of D1 receptors in the nucleus accumbens is associated with an increased phospho-Thr34-DARPP-32 expression after D1 receptor activation. These data suggest that an enduring increase in the sensitivity of the dopamine D1 receptor intracellular pathway sensitivity represents a neurobiological correlate associated with the development of locomotor sensitization to ethanol.

Citation: Possa Abrahao K, Oliveira Goeldner F, Souza-Formigoni MLO (2014) Individual Differences in Ethanol Locomotor Sensitization Are Associated with Dopamine D1 Receptor Intra-Cellular Signaling of DARPP-32 in the Nucleus Accumbens. PLoS ONE 9(6): e98296. doi:10.1371/journal.pone.0098296

Editor: Abraham A. Palmer, University of Chicago, United States of America

Received March 1, 2013; **Accepted** April 30, 2014; **Published** June 11, 2014

Copyright: © 2014 Abrahao et al. This is an open-access article distributed under the terms of the Creative Commons Attribution License, which permits unrestricted use, distribution, and reproduction in any medium, provided the original author and source are credited.

Funding: The work was supported by Fundação Amparo a Pesquisa do Estado de São Paulo (FAPESP - 2008/01819-5), Associação Fundo Incentivo a Pesquisa (AFIP), Conselho Nacional de de Desenvolvimento Científico e Tecnológico (CNPq) and Coordenadoria de Aperfeiçoamento de Pessoal de Nível Superior (CAPES). The funders had no role in study design, data collection and analysis, decision to publish, or preparation of the manuscript.

Competing Interests: The authors have declared that no competing interests exist.

* E-mail: mlosformigoni@unifesp.br

¤ Current address: Laboratory of Integrative Neuroscience, National Institute of Alcohol Abuse and Alcoholism/National Institutes of Health, Rockville, Maryland, United States of America.

Introduction

Although alcoholism is a worldwide problem resulting in millions of deaths_ENREF_1, only a small percentage of alcohol users become addicted [1]. Many studies have documented a marked heterogeneity in behavioral responsiveness to ethanol [2,3,4]. Psychomotor sensitization to ethanol, a form of drug-dependent behavioral adaptation (defined as a progressive increase in psychomotor stimulant response with repeated drug exposure), has been suggested as a behavioral marker for alcohol preference and/or abuse liability in both animals [5,6] and humans [7]. Our previous studies identified significant individual differences in the development of behavioral sensitization to ethanol in outbred Swiss Albino Webster mice. While a subgroup of ethanol-treated mice showed a robust sensitization (sensitized group), others, in spite of receiving identical ethanol treatment, failed to show this drug-induced behavioral plasticity (non-sensitized group) and presented similar levels of activity to a saline-treated control group [8,9]. Variations in the development of ethanol sensitization probably reflect individual differences in addiction vulnerability, since sensitized mice voluntarily drink more ethanol than non-sensitized or saline-treated control mice [10].

Drugs of abuse activate the mesolimbic dopamine reward system, promoting increased dopamine concentrations in the nucleus accumbens (NAc) [11,12], a brain region known to play an important role in drug reinforcement. Neuroadaptations in the NAc are supposed to mediate many of the behavioral changes that underlie addiction [13]. The released dopamine can act on both D1 and D2 subtypes of dopaminergic receptors. D1 dopamine receptors are coupled to stimulatory G-protein that stimulates adenylyl cyclase and activates cyclic AMP-dependent protein kinases such as the protein kinase A (PKA). PKA increases the phosphorylation of dopamine- and cAMP-regulated phosphoprotein of 32 kDa (DARPP-32) at threonine (Thr) 34 residue

(phospho-Thr34-DARPP-32). DARPP-32 is located in neurons containing dopamine receptors and plays a central role in dopaminergic [14] and glutamatergic signaling integrating the activity of these two pathways [15]. The dysfunction of these cellular pathways has been associated with several major neurologic and psychiatric disorders, including drug dependence [16].

Our previous data demonstrated that, after a long-term (two weeks) withdrawal following a 5-day ethanol treatment, those mice that developed sensitization showed functionally hyperresponsive D1 receptors in the NAc [8]. In the present study, we hypothesized that after a longer ethanol treatment (21 days), sensitized mice should present a more preeminent locomotor hyperresponsiveness to a D1 receptor agonist administration and that this could be associated with increased levels of phospho-Thr34-DARPP-32 by the activation of accumbal dopaminergic D1 receptors.

Materials and Methods

Subjects

All animal procedures were performed in accordance with the National Institutes of Health (NIH) Principles of Laboratory Animal Care (1985). The Committee for Ethics in Research of the Universidade Federal de São Paulo approved the protocol (CEP #0455/08). All procedures implemented in this study observed ethical criteria for minimizing the number of animals used and their suffering. A total of 139 male Swiss Albino Webster mice from the colony of CEDEME (Centro de Desenvolvimento de Modelos Experimentais para Medicina e Biologia - Universidade Federal de Sao Paulo) were housed in plastic cages (44×34×16 cm) in groups of 15–20 and given free access to food and water. Animals that underwent surgical procedures were housed in smaller plastic cages (30×19×13 cm) in groups of 4 or 5 after the surgery. They were kept in a temperature-controlled colony room (22°C±1°C) with lights on between 07:00 AM and 07:00 PM. Mice were approximately 75 days old at the beginning of each experiment.

Chronic ethanol treatment

To induce sensitization to the stimulant effects of ethanol, we adopted previously described procedures [8,17]. To assess baseline locomotor activity, we initially evaluated all the animals in one 15 min session without any drug treatment in Opto-Varimex cages (Columbus Instruments, Columbus, Ohio; 47.5×25.7×20.5 cm), which detect locomotor activity by interruptions of horizontal photoelectric beams. We compared the locomotor activity of the different treatment groups prior to the experiments to control the influence of baseline reactivity on treatment outcomes (please see Material S1). One day after baseline assessment, mice received either saline or 2.2 g/kg of ethanol (Synth, São Paulo, Brazil 15% w/v) daily during 21 days. On days 1, 7, 14 and 21 of the treatment, mice received saline or ethanol administration and were immediately placed in the locomotor activity cages, remaining there for 15 min. All the procedures were performed between 12:00 PM and 05:00 PM. According to their locomotor response on the 21st day test, the ethanol-treated mice were sorted and classified as sensitized mice (those whose activity levels were in the upper 33% of the distribution) or non-sensitized (those whose activity levels were in the lower 33% of the distribution), as described in previous studies [8,9,18]. The intermediate group of mice was not included in the experiments. This methodology allows studying the two extreme profiles of behavioral response to a same drug treatment and the possible factors involved in this individual variability.

Experiment 1 – D1 Receptor Activity on Locomotor Behavior

Surgical procedures

After the 21-day chronic ethanol treatment, we anesthetized the mice with xylazine (10 mg/kg in 0.01 ml/g, i.p.) and ketamine (8 mg/kg in 0.01 ml/g, i.p.) before placing them in a stereotactic apparatus (Model EFF-333, Insight Ltd., Brazil). We implanted bilateral stainless steel guide cannulae (23 gauge and 8.0 mm in length) 2.5 mm above the nucleus accumbens (NAc) (AP+1.2 mm, ML±1.0 mm, DV - 2.0 mm from bregma [19]. The guide cannulae were anchored to the skull with two additional stainless steel screws and dental cement. At the end of the surgery, stainless steel wire stylets were inserted into the guide cannulae to prevent occlusion. The mice were allowed to recover for 5–10 days. In the challenge tests, drugs were infused bilaterally into the NAc using 10.5-mm-long injection cannulae (30 gauge) that extended an additional 2.5 mm below the guide cannulae tips. The injectors were connected via polyethylene microbore tubing to 10- µl Hamilton microsyringes mounted on a micro-drive pump (Model EFF-311, Insight Ltd., Brazil). Each microinjection was performed in a volume of 0.2 µl per side at a rate of 0.2 µl/min. Thirty seconds after the infusion, the injection cannulae were removed, and the animals were placed in the activity cages.

Challenges

After the chronic treatment with ethanol or saline, different subgroups of mice of saline, non-sensitized and sensitized groups (one group for each dose of D1 receptor agonist) were initially challenged with intra-NAc administration of saline and, 48 h later, submitted to a second challenge with 0.1 or 1 µg/side of SKF (D1 receptor agonist SKF-38393 hydrochloride, Sigma-Aldrich, Brazil), in order to assess the D1 receptor function in sensitized or non-sensitized mice to the stimulant effects of ethanol.

Histology

After the challenges, the mice were anesthetized with a high dose of ketamine and euthanized by decapitation. Their brains were removed, frozen on dry ice and stored at −80°C. We analyzed the placements of the injection sites, according to the atlas of Paxinos and Franklin [19], by histological examination of frozen 40- µm coronal brain sections stained with cresyl violet. Only the data from mice whose cannulae placements were correct (see Figure 1) were included in the analysis. We lost eleven mice from the saline group, eighteen mice from the non-sensitized group and seventeen mice from the sensitized group due to incorrect placements or other problems during the surgery.

Experiment 2 – D1 Receptor Activity on Phosphorylation of DARPP-32

Western blot

Fourteen days after the chronic ethanol treatment protocol, mice from saline, non-sensitized and sensitized groups received an i.p. administration of 10 mg/kg SKF and, 20 min later, they were euthanized by decapitation. Brains were quickly removed, frozen over dry ice and stored at −80°C. The nucleus accumbens (bregma 1.94 to 0.62 mm) were punched from the brain slices sectioned in the coronal plane on a Hacker-Bright cryostat at −20°C, according to the Paxinos and Franklin mouse Atlas [19]. Total protein extract was prepared using lysis buffer containing 50 mM Tris-HCl (pH 7.4), 150 mM NaCl, 1% Triton x-100, 0.1% SDS, 1 mM EDTA, and 1% sodium deoxycholate. The protein concentration was determined using the Bio-Rad Protein

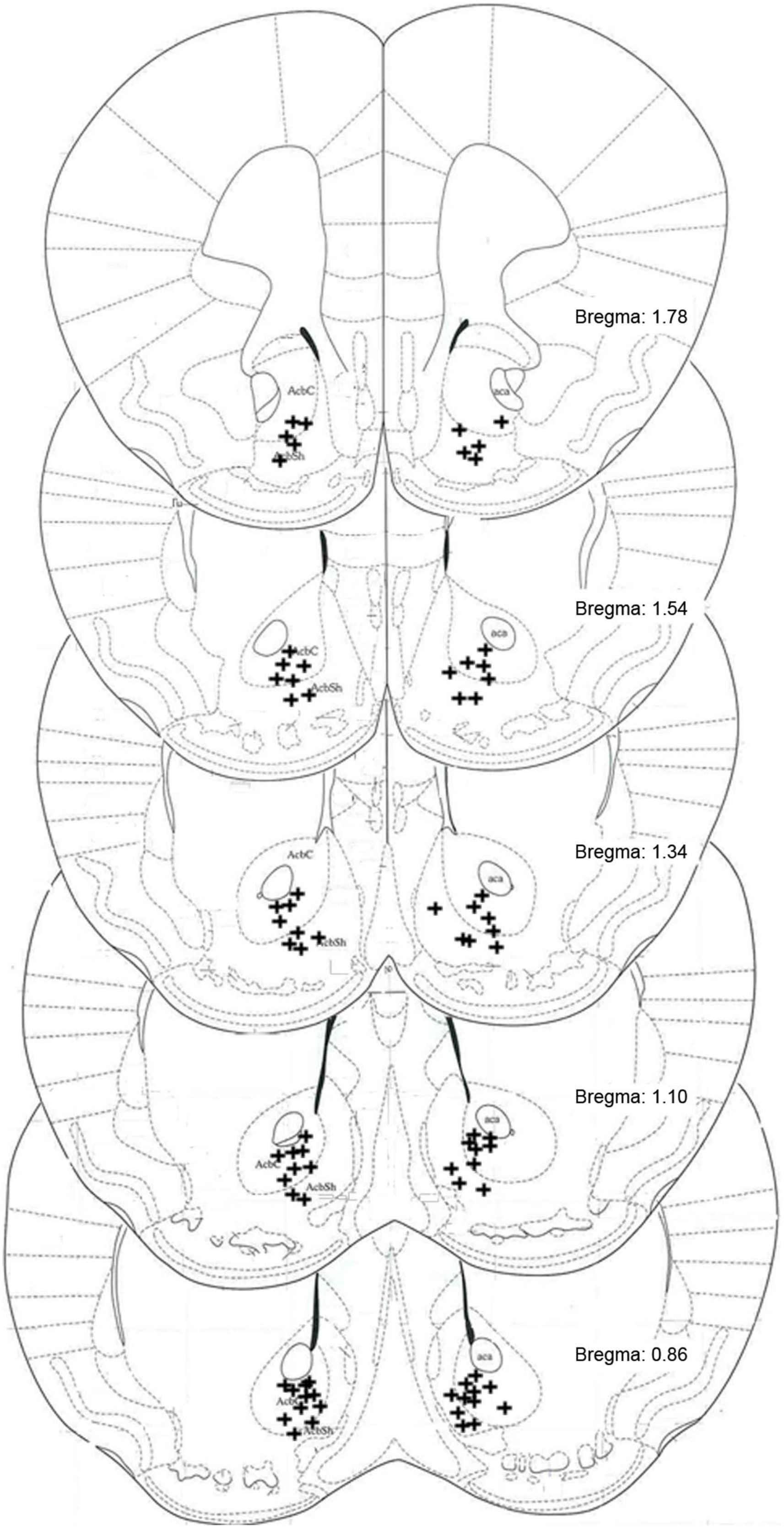

Figure 1. Diagram of approximate microinjections hits. The crosses (+) represent the positions of microinjections in both sides of the Nucleus Accumbens considered correct. The basic diagram is a modified representation of Paxinos and Franklin (2004) atlas.
doi:10.1371/journal.pone.0098296.g001

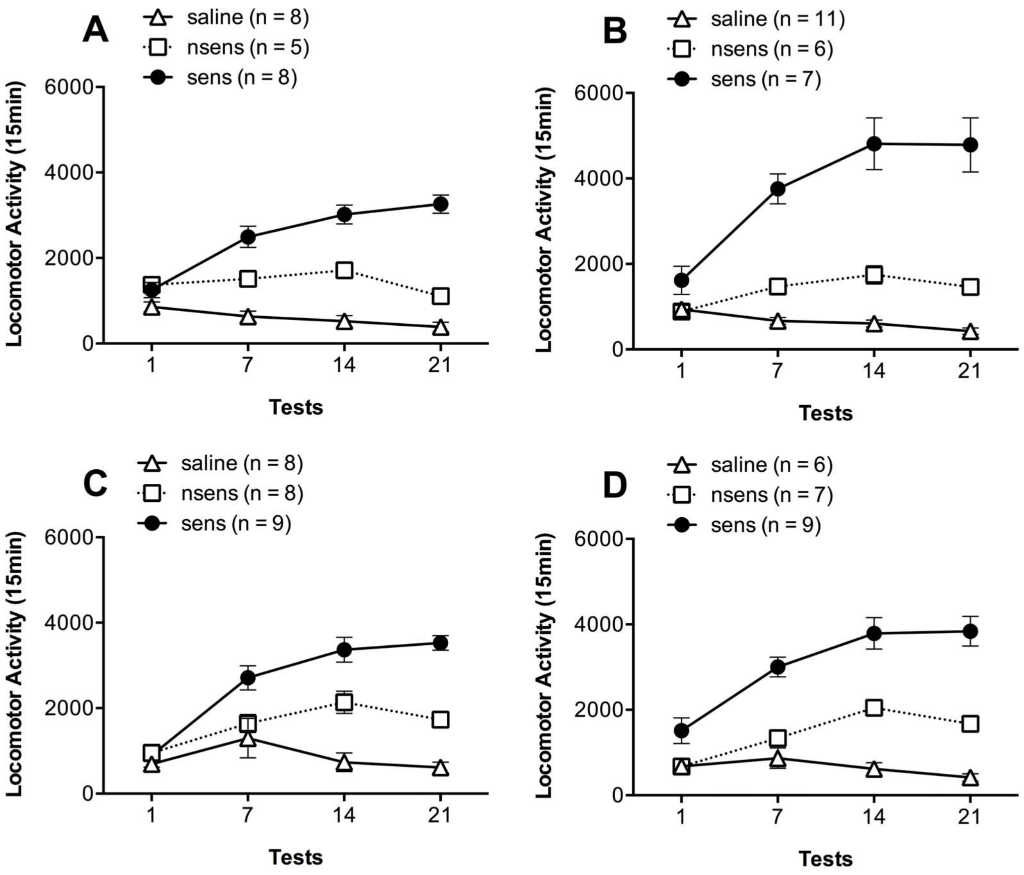

Figure 2. There is clear individual variability to the development of sensitization to ethanol. Locomotor activity for 15 min (means ± S.E.M.) of mice treated with saline or 2.2 g/kg ethanol i.p. (classified as non-sensitized (nsens) or sensitized (sens) based on their locomotor activity in the 21st day test) in the tests performed on days 1, 7, 14 and 21. (**A**) Cohort of mice (saline, n = 8; nsens, n = 5; sens, n = 8) that received the lower dose of SKF during the challenge phase. (**B**) Cohort of mice (saline, n = 11; nsens, n = 6; sens, n = 7) that received the higher dose of SKF during the challenge phase. (**C**) Cohort of mice (saline, n = 8; nsens, n = 8; sens, n = 9) that was designed for DARPP-32 measures after saline administration. (**D**) Cohort of mice (saline, n = 6; nsens, n = 7; sens, n = 7) that was designed for DARPP-32 measures after SKF administration. * indicates significantly higher activity levels than those presented by the saline and nsens groups during the same test ($p<0.05$) and when compared to their own levels in test 1 ($p<0.05$).
doi:10.1371/journal.pone.0098296.g002

Assay – Hercules, CA [20]. 50 ìg proteins were separated by 10% SDS-polyacrylamide gel electrophoresis and transferred to a nitrocellulose membrane at 100 volts for 60 min. The blots were blocked with 1% bovine serum albumin in TBST (10 mM Tris, pH 7.5, 100 mM NaCl and 0.1% Tween 20) for 1 hour at room temperature and then incubated with the rabbit polyclonal antibody against DARPP-32 (Abcam, Cambridge, MA) and phospho-DARPP-32 (Santa Cruz Biotechnology, Santa Cruz, CA) in 1:1000 dilution in 1% bovine serum albumin/TBST overnight at 4°C. Excess of primary antibody was removed with three washes with TBST prior to 1 hour incubation at room temperature with goat anti-rabbit secondary antibody conjugated with alkaline phosphatase (Santa Cruz Biotechnology, Santa Cruz, CA) in 1:5000 dilution in 5% nonfat dry milk/TBST for 1 hour. Membrane was incubated for 5 min in substrate for the detection of the alkaline phosphatase (Sigma Fast BCIP/NBT - Sigma Aldrich Inc. St. Louis). The bands were analyzed and quantitation was done using AlphaEaseFC Software (Alpha Innotech, San Leandro, CA) with â-tubulin as an endogenous reference. The assay was conducted in duplicate for each sample. Samples of total DARPP-32 and phospho-DARPP-32 were harvested separated.

Data analyses

For each experiment, the locomotor activity counts recorded during the treatment or challenge tests were evaluated by two-way analysis of variance (ANOVA) for repeated measures, with group (saline, sensitized or non-sensitized mice) as the independent factor and the days of the tests, time during the challenge or different challenges as the repeated measure factors. The comparison of the levels of expression of total DARPP-32, phospho-Thr34-DARPP-32 and phospho-Thr34-DARPP-32/total DARPP-32 among saline, sensitized or non-sensitized mice was made by one-way ANOVAs. Newman-Keuls tests for multiple comparisons were used for *post-hoc* analyses. The level of significance was set at 5% for all analyses. All analyses were made using the software Statistica version 10 (Statsoft Inc, 2011).

Results

Development of behavioral sensitization to ethanol

Figure 2A-B shows the development of behavioral sensitization to the stimulant effects of ethanol in the two cohorts of mice that received 0.1 μg/side or 1.0 μg/side of SKF in the pharmacological phase of Experiment 1. In both cohorts, two clearly different profiles of locomotor activity were observed: a group that

developed a clear sensitization after the tests (sensitized mice) and another group that presented low activity levels (non-sensitized mice). The latter presented similar levels to those observed in the control (saline) group. In Figure 2A, a repeated measures ANOVA revealed significant effects of group ($F(2,15) = 72.46$; $p<0.001$), test ($F(3,45) = 10.21$; $p<0.001$) and group-test interaction factors ($F(6,45) = 20.23$; $p<0.001$). The sensitized group of mice showed robust behavioral sensitization with a progressive increase in the activity levels during the ethanol treatment ($p<0.05$). They presented significantly higher activity levels on tests 14 and 21 than on tests 1 and 7; and higher levels than those from saline and non-sensitized mice on tests 7, 14 and 21 ($p<0.05$). The non-sensitized mice did not display progressive locomotor stimulation but similar activity levels to those observed in the control group. It is noteworthy that there were no significant differences in the acute (test 1) locomotor activity levels among saline, sensitized and non-sensitized groups. Similar results were observed as regards the cohort that would receive 1.0 μg/side of SKF as shown in Figure 2B (repeated measures ANOVA: effects of group $F(2,21) = 50.65$; $p<0.001$, test $F(3,63) = 32.24$; $p<0.001$ and group-test interaction factors $F(6,63) = 29.34$; $p<0.001$).

Figure 2C–D shows the development of behavioral sensitization to the stimulant effects of ethanol in mice that received saline or SKF-38393 prior to the measures of expression of DARPP-32 and phosphoDARPP-32. Similar results were observed for both groups of mice which received saline or SKF-38393 administration prior to the euthanasia (repeated measures ANOVA: Figure 2C effects of group $F(2,44) = 27.73$; $p<0.001$, test $F(3,66) = 28.11$; $p<0.001$ and group-test interaction factors $F(6,66) = 12.17$; $p<0.001$. Figure 2D shows the effects of group $F(2,19) = 52.28$; $p<0.001$, test $F(3,57) = 15.33$; $p<0.001$ and group-test interaction factors $F(6,57) = 7.04$; $p<0.001$). After *post-hoc* analyses, we observed similar results to those described in the previous experiment.

It is important to consider that the development of behavioral sensitization could be associated with the initial baseline locomotor response to novel environment. To address whether the baseline levels could predict which mice would be categorized as sensitized versus non-sensitized, we retrospectively analyzed the baseline (novelty) data of the three groups of animals: saline, non-sensitized and sensitized mice in the four cohorts used in the present study (Material S1). No differences were observed in the locomotor levels among the groups when the animals were exposed to the locomotor activity cage for the very first time (Figure S1). This indicates that the baseline locomotor activity levels do not predict the development of behavioral sensitization to the stimulant effect of ethanol.

Functionally hyperresponsive D1 receptors in ethanol sensitized mice

The pharmacological phase of this study was performed to compare sensitized and non-sensitized mice regarding their D1 dopamine receptors responsiveness to intra-NAc administration of SKF in different doses. Figure 3A–B shows the locomotor activity levels of saline, non-sensitized and sensitized groups that received saline and, 48 h later, 0.1 μg/side of SKF. We performed repeated measures ANOVA for each challenge, considering group as the independent factor and the locomotor activity along with time as the dependent variable. In the saline challenge (Figure 3A), the ANOVA revealed significant effect of the time factor ($F(15,240) = 20.76$; $p<0.001$), but not of the group ($F(2,16) = 1.49$) or the group-time interaction ($F(30,240) = 1.06$) factors. We observed a decrease in the locomotor activity levels during time ($p<0.05$). In the 0.1 μg/side SKF challenge (Figure 3B), the ANOVA revealed significant effect of time factor ($F(15,240) = 12.64$; $p<0.001$), but no effect of group factor ($F(2,16) = 1.69$) or group-time interaction ($F(30,240) = 1.43$). Despite no group effect, we observed a slight increase in the locomotor activity of the sensitized group during the first 15 minutes after the agonist administration. To analyze this effect, we compared the total locomotor activity levels during the first 15 minutes after saline and 0.1 μg/side SKF challenges (see Figure 3C). The ANOVA revealed significant effect of challenge factor ($F(1,16) = 26.25$; $p<0.001$) but not of group factor ($F(2,16) = 2.81$; $p = 0.09$) or group-challenge interaction ($F(2,16) = 3.06$; $p = 0.07$). The unprotected *post-hoc* analyses revealed that sensitized mice showed higher levels of locomotor activity after 0.1 μg/side SKF administration than after saline challenge. Besides, in the 0.1 μg/side SKF challenge, sensitized mice showed higher levels of locomotor activity than the other groups.

Figure 3D–F shows the locomotor activity levels of saline, non-sensitized and sensitized groups that received saline and, 48h later, 1.0 μg/side of SKF. In the saline challenge (Figure 3D), the ANOVA did not detect the factor group as significant ($F(2,21) = 3.56$), but revealed significant effect of time ($F(11,231) = 2,09$; $p<0,05$) and no group-time interaction ($F(22,231) = 1,3$). We observed a decrease in the locomotor activity during time ($p<0.05$). In the 1.0 μg/side SKF challenge (Figure 3E), the ANOVA revealed significant effect of the time factor ($F(11,231) = 12.13$; $p<0.001$), group ($F(2,21) = 6.07$), but no group-time interaction ($F(22,231) = 1.26$) factors. The close analyze of the graph suggests that the sensitized group had higher stimulant effect than the others. Because of this, the total locomotor activity levels during the first 60 minutes after saline or 1.0 μg/side SKF challenges are presented in Figure 3F. The ANOVA revealed significant effect of challenge factor ($F(1,21) = 41.79$; $p<0.001$), group ($F(2,21) = 6.19$; $p<0.05$) and group-challenge interaction ($F(2,16) = 5.67$; $p<0.05$) factors. The *post-hoc* analyses revealed that after 1.0 μg/side SKF intra-NAc administration, sensitized mice showed higher levels of locomotor activity than in the saline challenge and higher levels than the other two groups: saline and non-sensitized.

Increased phosphorylation of DARPP-32 in ethanol sensitized mice

Figure 4 shows the protein expression (% of controls) of total DARPP-32, phospho-Thr34-DARPP-32 and phospho-Thr34-DARPP-32/total DARPP-32 in the NAc, 14 days after saline or ethanol treatment. No differences were observed among the three groups in the total DARPP-32 expression after saline administration (Figure 4A; $F(2,22) = 2.12$). However, non-sensitized and sensitized groups had lower levels of phospho-Thr34-DARPP-32 expression than saline-treated controls (Figure 4A, $F(2,22) = 54.00$; $p<0.001$). We observed lower phospho-Thr34-DARPP-32/total DARPP-32 expression in non-sensitized and sensitized groups when compared to saline group ($F(2,22) = 197.58$; $p<0.001$).

As seen in Figure 4B, after SKF administration the non-sensitized and sensitized groups showed higher levels of expression of DARPP-32 in the NAc when compared to the levels of saline treated mice that have previously received SKF ($p<0.05$). The dopaminergic D1 agonist administration induced higher levels of phospho-Thr34-DARPP-32 expression in the sensitized group than in the saline or non-sensitized groups ($F(2,17) = 173.62$; $p<0.001$). Besides, the non-sensitized group showed higher levels of phospho-Thr34-DARPP-32 expression than the saline controls ($p<0.05$). After D1 agonist administration, we observed that phospho-Thr34-DARPP-32/total DARPP-32 expression was

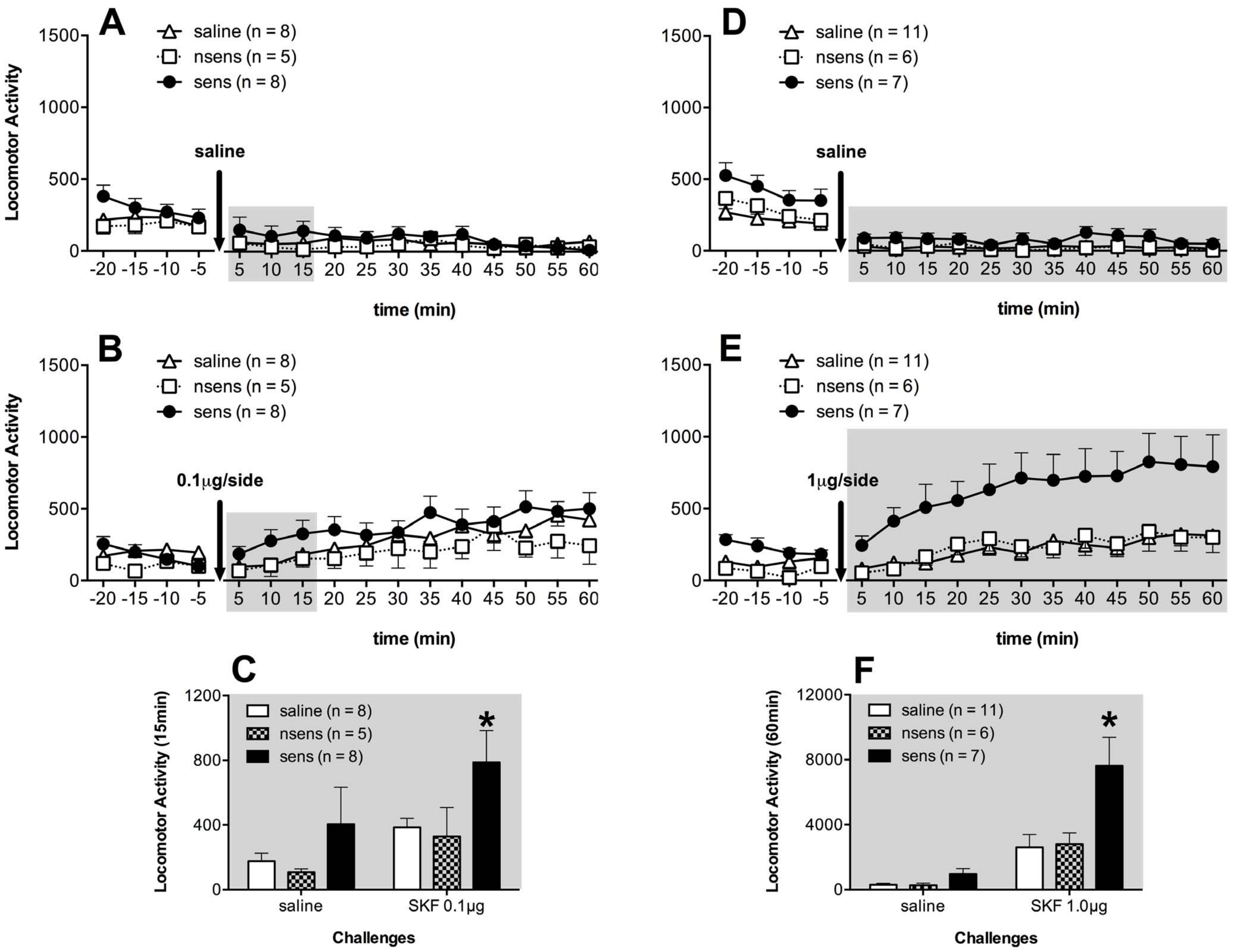

Figure 3. D1 receptor agonist induced hyperresponsive locomotion in sensitized mice. Locomotor activity (means + S.E.M.) of the saline, sens and nsens in the challenges with intra-NAc administration of saline and SKF-38393 at 0.1 or 1 µg/side. Each challenge was performed 48 h after the previous one. (**A**) Locomotor activity 20 min before and 60 min after saline intra-NAc administration of saline (n = 8), nsens (n = 5) and sens (n = 8) groups. (**B**) Locomotor activity 20 min before and 60 min after SKF 0.1 µg/side intra-NAc administration of saline (n = 8), nsens (n = 5) and sens (n = 8) groups, 48 h after the saline challenge (A). (**C**) Locomotor activity during 15 min (gray backgrounds in figures A and B), after intra-NAc administration of saline and SKF 0.1 µg/side. (**D**) Locomotor activity 20 min before and 60 min after saline intra-NAc administration of saline (n = 11), nsens (n = 6) and sens (n = 7) groups. (**E**) Locomotor activity 20 min before and 60 min after SKF 1 µg/side intra-NAc administration of saline (n = 11), nsens (n = 6) and sens (n = 7) groups, 48 h after the saline challenge (D). (**F**) Locomotor activity during 60 min (gray backgrounds in figures D and E), after intra-NAc administration of saline and SKF 1 µg/side. * indicates significantly higher activity levels than those of the saline and nsens mice and higher locomotor activity levels than their own levels in the saline challenge ($p<0.05$).
doi:10.1371/journal.pone.0098296.g003

higher in the sensitized mice than in the non-sensitized or saline mice ($F(2,17) = 235.35$; $p<0.001$).

Discussion

We demonstrated an important association between the variability in behavioral sensitization to the stimulant effect of ethanol and the functionality of dopamine D1 receptors and its intra-cellular cascade pathway. After a two-week drug-free period, following a 21-day ethanol treatment, those mice that had developed high levels of sensitization showed an increased locomotor response and an increased phosphorylation of DARPP-32 in NAc after the administration of a D1 receptor agonist. It is important to note that these effects were not observed in all mice submitted to the 21-day ethanol treatment. In spite of receiving the same amount of ethanol, non-sensitized mice did not present alterations in the functionality of the accumbal D1 receptors when compared to controls.

Repeated exposure to drugs of abuse, such as ethanol, progressively increases their psychomotor stimulant effects, a phenomenon known as behavioral sensitization [13,21,22]. Some authors have proposed that sensitization to drugs of abuse can be used as an indirect measure of the neural adaptations related to the transition from controlled, casual use to compulsive drug use and addiction [23,24]. Steketee and Kalivas consider that there is similarity between the neural circuitry and the drug-induced neurochemical changes involved in the process of sensitization and reinstatement of drug use. As regards drug use and addiction, there is important variability in the behavioral responses to chronic drug treatment [25]. As demonstrated in previous studies, outbred Swiss Albino mouse strain show significant inter-individual variability in the development of behavioral sensitiza-

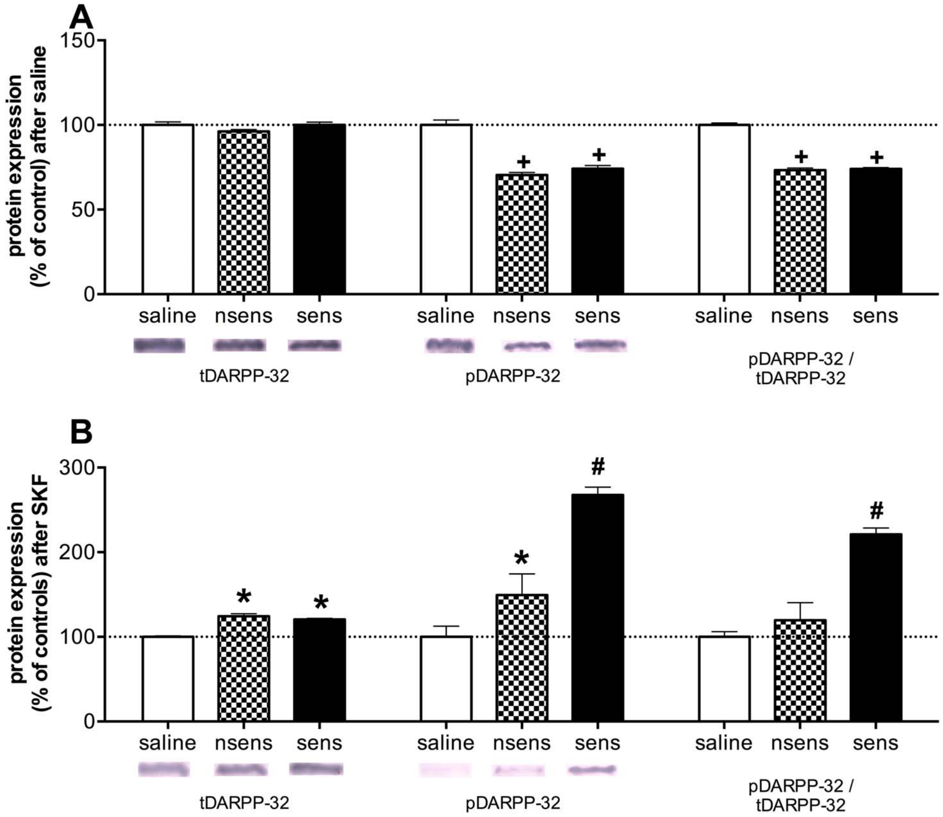

Figure 4. D1 receptor agonist induced accumbal DARPP-32 hyperphosphorylation in sensitized mice. (**A**) Protein expression of total DARPP-32, phospho-Thr34-DARPP-32 and phospho-Thr34-DARPP-32/total DARPP-32 in the NAc in saline (n = 8), nsens (n = 8) and sens (n = 9) groups, 20 min after i.p. saline administration. (**B**) Protein expression of total DARPP-32, phospho-Thr34-DARPP-32 and phospho-Thr34-DARPP-32/total DARPP-32 in the NAc in saline (n = 6), nsens (n = 7) and sens (n = 7) groups, 20 min after 10 mg/kg i.p. SKF-38393 administration. * indicates significantly higher levels than saline and nsens groups ($p<0.05$). + indicates significantly lower levels than saline group ($p<0.05$). # indicates significantly higher levels than saline and nsens groups ($p<0.05$).
doi:10.1371/journal.pone.0098296.g004

tion to the stimulant effect of ethanol [8,9,17,18,26,27]. We demonstrated that after two weeks of a drug-free period, the sensitized, but not the non-sensitized mice, consumed ethanol in a significantly higher amount than the saline controls [10]. Considering the above-mentioned findings, we hypothesize that sensitized mice could be more vulnerable to alcohol addiction. Since the neuroadaptations observed after ethanol treatment may be associated with an increased vulnerability to addiction or relapse, it is important to understand their association with the variability in the behavioral response to ethanol treatment.

The increase of dopamine release in NAc observed after ethanol administration [28] may be potentiated by chronic treatment. After a 24-hour withdrawal from ethanol chronic treatment, there is no difference in the D1 receptor binding among sensitized, non-sensitized and saline groups [18]. However, it is possible that neural changes may counterpoise the lack of dopamine activation after a long-term withdrawal. In a previous study, we demonstrated that, after a two-week withdrawal period which followed a 5-day ethanol treatment, sensitized mice presented functionally hyper responsive D1 receptors in the NAc [8]. The present data corroborated and extended this conclusion, pointing out that a longer ethanol treatment (21 days) may potentiate the D1 receptor neural adaptation. Both doses of the D1 receptor agonist, when administered into the NAc, induced a more robust increase of the locomotor activity in sensitized mice than in non-sensitized or saline ones. We have also demonstrated the presence of significant hyperfunctional accumbal D2 receptors in sensitized mice after two weeks of withdrawal from the ethanol treatment [27]. We could consider the hyperfunctionality of the dopaminergic receptors in the nucleus accumbens is a response that counterpoised a hypodopaminergia syndrome associated with ethanol long-term withdrawal. While the hypodopaminergia may contribute to relapse, the hyperfunctionality of dopaminergic receptors seems to be associated with the increased behavioral response to drugs.

The DARPP-32 activity seems to be an important factor in the ethanol reinforcement properties, since DARPP-32 knock-out mice drank less ethanol, did not develop conditioned place preference and had increased sensitivity to the ethanol stimulant effect [29]. Besides, DARPP-32 phosphorilation is regulated by ethanol [30] and differences in the regulation of this molecule contribute to different ethanol drinking patterns in rats [31]. Here we demonstrated that after a withdrawal period following a chronic ethanol treatment, sensitized and non-sensitized mice presented lower levels of phospho-Thr34-DARPP-32 expression than saline treated controls, which may indicate low activity of endogenous dopamine in animals pre-treated with ethanol. However, after the D1 receptor agonist administration, ethanol sensitized and non-sensitized mice did present higher levels of DARPP-32 than the saline control group that also received D1 agonist administration. The interpretation of these data is not obvious considering the specific transcriptional mechanisms that

regulate striatal DARPP-32 expression remain enigmatic. It's known, however, that some factors can modulate DARPP-32 transcription. We may speculate that in ethanol-treated mice these mechanisms could be hypersensitive and mediate DARPP-32 expression after the D1 agonist administration only in those mice that had previously received chronic ethanol treatment. We also observed that sensitized mice had higher levels of phospho-Thr34-DARPP-32 expression than non-sensitized or saline ones. These results corroborate the assumption that DARPP-32 plays a key role in the development of D1 receptors involved in motor stimulatory effects [32]. Therefore, the locomotor hyperresponsiveness to the accumbal direct-acting D1 receptor agonist observed in sensitized mice seems to be associated with the increase in DARPP-32 phosphorilation at threonine 34.

Phospho-Thr34-DARPP-32 is a potent inhibitor of protein phosphastase -1 (PP-1). This cascade increases the phosphorylation of the major subunit of the glutamatergic NMDA receptor, NR1 [15,33]. As a consequence, the activation of D1 receptors increases the phosphorylation of NMDA receptors through DARPP-32 pathway. It was demonstrated that the phosphorylation of the NR1 subunit of NMDA receptors strongly decreases the acute ethanol inhibition of NMDA receptors [30]. However, after a long-term withdrawal from chronic ethanol treatment, diverse neuroadaptations in NMDA receptors can be observed. We showed that, after a two-week withdrawal period, ethanol sensitized mice had a decrease in the functionality and expression of NMDA receptor in the NAc [10]. The manipulation of DARPP-32 activity could contribute to modulate the deficit in NMDA functionality. As mention by others, DARPP-32 could be considered a potential therapeutic target for the treatment of alcohol or other drugs addiction, as well as for several other psychiatric disorders [34].

The different levels of accumbal D1 receptor/DARPP-32 signaling function observed in mice with different profiles in ethanol sensitization may be an important biomarker of behavioral adaptations observed during ethanol administration. Indeed, non-sensitized mice, which did not develop sensitization, did not differ from controls in relation to D1 receptor agonist induced locomotion and DARPP-32 phosphorylation, reinforcing the relation between the behavioral and neuronal adaptations. Considering that behavioral sensitization may be associated with the reinstatement of drug use, the functionality of the intracellular pathway of D1 receptors could contribute to the vulnerability to relapse. A better understanding of the molecular substrates responsible for the different levels of sensitization could unravel new targets for the development of more effective therapies for ethanol abuse.

Supporting Information

Figure S1 Novelty response does not predict the development of ethanol locomotor sensitization. Locomotor activity (means ± S.E.M.) for 15 min in the novelty test when saline, non-sensitized (nsens) or sensitized (sens) groups were exposure to the locomotor activity cage for the first time without drug administrations of mice. (**A**) Cohort of mice (saline, n = 8; nsens, n = 5; sens, n = 8) that received the lower dose of SKF during the challenge phase. (**B**) Cohort of mice (saline, n = 11; nsens, n = 6; sens, n = 7) that received the higher dose of SKF during the challenge phase. (**C**) Cohort of mice (saline, n = 8; nsens, n = 8; sens, n = 9) that was designed for DARPP-32 measures after saline administration. (**D**) Cohort of mice (saline, n = 6; nsens, n = 7; sens, n = 7) that was designed for DARPP-32 measures after SKF administration.
(DOCX)

Material S1 Statistical analyses of novelty response for each behavioral sensitization experiment.
(DOCX)

Acknowledgments

We thank Valdegar Xavier de Oliar and João Victor Nicodemos Pildervasser for their technical support during the experiments and Maria Helena Pagdi for the language review.

Author Contributions

Conceived and designed the experiments: KPA MLOSF. Performed the experiments: KPA FOG. Analyzed the data: KPA MLOSF. Contributed reagents/materials/analysis tools: KPA FOG MLOSF. Wrote the paper: KPA MLOSF.

References

1. Rehm J, Mathers C, Popova S, Thavorncharoensap M, Teerawattananon Y, et al. (2009) Global burden of disease and injury and economic cost attributable to alcohol use and alcohol-use disorders. Lancet 373: 2223–2233.
2. Bell RL, Rodd ZA, Lumeng L, Murphy JM, McBride WJ (2006) The alcohol-preferring P rat and animal models of excessive alcohol drinking. Addict Biol 11: 270–288.
3. Melon LC, Boehm SL 2nd (2011) Role of genotype in the development of locomotor sensitization to alcohol in adult and adolescent mice: comparison of the DBA/2J and C57BL/6J inbred mouse strains. Alcohol Clin Exp Res 35: 1351–1360.
4. Fidler TL, Dion AM, Powers MS, Ramirez JJ, Mulgrew JA, et al. (2011) Intragastric self-infusion of ethanol in high- and low-drinking mouse genotypes after passive ethanol exposure. Genes Brain Behav 10: 264–275.
5. Lessov CN, Palmer AA, Quick EA, Phillips TJ (2001) Voluntary ethanol drinking in C57BL/6J and DBA/2J mice before and after sensitization to the locomotor stimulant effects of ethanol. Psychopharmacology (Berl) 155: 91–99.
6. Grahame NJ, Rodd-Henricks K, Li TK, Lumeng L (2000) Ethanol locomotor sensitization, but not tolerance correlates with selection for alcohol preference in high- and low-alcohol preferring mice. Psychopharmacology (Berl) 151: 252–260.
7. Newlin DB, Thomson JB (1999) Chronic tolerance and sensitization to alcohol in sons of alcoholics: II. Replication and reanalysis. Exp Clin Psychopharmacol 7: 234–243.
8. Abrahao KP, Quadros IM, Souza-Formigoni ML (2011) Nucleus accumbens dopamine D receptors regulate the expression of ethanol-induced behavioural sensitization. Int J Neuropsychopharmacol 14: 175–185.
9. Souza-Formigoni ML, De Lucca EM, Hipolide DC, Enns SC, Oliveira MG, et al. (1999) Sensitization to ethanol's stimulant effect is associated with region-specific increases in brain D2 receptor binding. Psychopharmacology (Berl) 146: 262–267.
10. Abrahao KP, Oriwodola OJ, Rau AR, Skelly MJ, Carter E, et al. (2013) Locomotor sensitization to ethanol impairs NMDA receptor-dependent synaptic plasticity in the nucleus accumbens and increases ethanol self-administration. The Journal of Neuroscience, in press.
11. Di Chiara G (1999) Drug addiction as dopamine-dependent associative learning disorder. Eur J Pharmacol 375: 13–30.
12. Diana M, Gessa GL, Rossetti ZL (1992) Lack of tolerance to ethanol-induced stimulation of mesolimbic dopamine system. Alcohol Alcohol 27: 329–333.
13. Vanderschuren LJ, Kalivas PW (2000) Alterations in dopaminergic and glutamatergic transmission in the induction and expression of behavioral sensitization: a critical review of preclinical studies. Psychopharmacology (Berl) 151: 99–120.
14. Fienberg AA, Hiroi N, Mermelstein PG, Song W, Snyder GL, et al. (1998) DARPP-32: regulator of the efficacy of dopaminergic neurotransmission. Science 281: 838–842.
15. Snyder GL, Fienberg AA, Huganir RL, Greengard P (1998) A dopamine/D1 receptor/protein kinase A/dopamine- and cAMP-regulated phosphoprotein (Mr 32 kDa)/protein phosphatase-1 pathway regulates dephosphorylation of the NMDA receptor. J Neurosci 18: 10297–10303.
16. Svenningsson P, Nairn AC, Greengard P (2005) DARPP-32 mediates the actions of multiple drugs of abuse. AAPS J 7: E353–360.
17. Abrahao KP, Quadros IM, Souza-Formigoni ML (2009) Individual differences to repeated ethanol administration may predict locomotor response to other drugs, and vice versa. Behav Brain Res 197: 404–410.
18. Quadros IM, Nobrega JN, Hipolide DC, de Lucca EM, Souza-Formigoni ML (2002) Differential propensity to ethanol sensitization is not associated with

altered binding to D1 receptors or dopamine transporters in mouse brain. Addict Biol 7: 291–299.
19. Paxinos G, Franklin KBJ (2004) The Mouse Brain. San Diego: Elsevier.
20. Bradford MM (1976) A rapid and sensitive method for the quantitation of microgram quantities of protein utilizing the principle of protein-dye binding. Anal Biochem 72: 248–254.
21. Masur J, Oliveira de Souza ML, Zwicker AP (1986) The excitatory effect of ethanol: absence in rats, no tolerance and increased sensitivity in mice. Pharmacol Biochem Behav 24: 1225–1228.
22. Segal DS, Mandell AJ (1974) Long-term administration of d-amphetamine: progressive augmentation of motor activity and stereotypy. Pharmacol Biochem Behav 2: 249–255.
23. Robinson TE, Berridge KC (1993) The neural basis of drug craving: an incentive-sensitization theory of addiction. Brain Res Brain Res Rev 18: 247–291.
24. Vanderschuren LJ, Schoffelmeer AN, Mulder AH, De Vries TJ (1999) Dopaminergic mechanisms mediating the long-term expression of locomotor sensitization following pre-exposure to morphine or amphetamine. Psychopharmacology (Berl) 143: 244–253.
25. Steketee JD, Kalivas PW (2011) Drug wanting: behavioral sensitization and relapse to drug-seeking behavior. Pharmacol Rev 63: 348–365.
26. Masur J, dos Santos HM (1988) Response variability of ethanol-induced locomotor activation in mice. Psychopharmacology (Berl) 96: 547–550.
27. Abrahao KP, Quadros IM, Andrade AL, Souza-Formigoni ML (2011) Accumbal dopamine D2 receptor function is associated with individual variability in ethanol behavioral sensitization. Neuropharmacology.
28. Yim HJ, Gonzales RA (2000) Ethanol-induced increases in dopamine extracellular concentration in rat nucleus accumbens are accounted for by increased release and not uptake inhibition. Alcohol 22: 107–115.
29. Risinger FO, Freeman PA, Greengard P, Fienberg AA (2001) Motivational effects of ethanol in DARPP-32 knock-out mice. J Neurosci 21: 340–348.
30. Maldve RE, Zhang TA, Ferrani-Kile K, Schreiber SS, Lippmann MJ, et al. (2002) DARPP-32 and regulation of the ethanol sensitivity of NMDA receptors in the nucleus accumbens. Nat Neurosci 5: 641–648.
31. Nuutinen S, Kiianmaa K, Panula P (2011) DARPP-32 and Akt regulation in ethanol-preferring AA and ethanol-avoiding ANA rats. Neurosci Lett 503: 31–36.
32. Scott L, Forssberg H, Aperia A, Diaz-Heijtz R (2005) Locomotor effects of a D1R agonist are DARPP-32 dependent in adult but not weanling mice. Pediatr Res 58: 779–783.
33. Hemmings HC Jr, Greengard P, Tung HY, Cohen P (1984) DARPP-32, a dopamine-regulated neuronal phosphoprotein, is a potent inhibitor of protein phosphatase-1. Nature 310: 503–505.
34. Reis HJ, Rosa DV, Guimaraes MM, Souza BR, Barros AG, et al. (2007) Is DARPP-32 a potential therapeutic target? Expert Opin Ther Targets 11: 1649–1661.
